# A Remedy in Nervous Disorders When Characterized by Melancholia

**Published:** 1897-06

**Authors:** 


### A REMEDY IN NERVOUS DISORDERS WHEN CHARACTERIZED BY MELANCHOLIA.

The "Reference Book of Practical Therapeutics," by Frank P. Foster, M.D., Editor of *The New York Medical Journal*, which has recently been issued by D. Appleton & Co., of New York

City, contains an article of which the following is an excerpt, which we feel expresses the consensus of medical opinion as adduced by actual results: "Antikamnia is an American preparation that has come into extensive use as an analgetic and antipyretic. It is a white, crystalline, odorless powder, having a slightly aromatic taste, soluble in hot water, almost insoluble in cold water, but more fully soluble in alcohol. * * *

"As an antipyretic it acts rather more slowly than antipyrine or acetanilid, but efficiently, and it has the advantage of being free, or almost free from any depressing effect on the heart. Some observers even think that it exerts a sustaining action on the circulation. As an analgetic it is characterized by promptness of action and freedom from the disagreeable effects of the narcotics. It has been much used, and with very favorable results in neuralgia, influenza, and various nervous disorders characterized by melancholia. The dose of antikamnia is from three to ten grains, and it is most conveniently given in the form of tablets."

